# Comprehensive Characterization of Transcriptional Activity during Influenza A Virus Infection Reveals Biases in Cap-Snatching of Host RNA Sequences

**DOI:** 10.1128/JVI.01720-19

**Published:** 2020-05-04

**Authors:** Sara Clohisey, Nicholas Parkinson, Bo Wang, Nicolas Bertin, Helen Wise, Andru Tomoiu, FANTOM5 Consortium, Kim M. Summers, Ross W. Hendry, Piero Carninci, Alistair R. R. Forrest, Yoshihide Hayashizaki, Paul Digard, David A. Hume, J. Kenneth Baillie

**Affiliations:** a Division of Genetics and Genomics, The Roslin Institute, University of Edinburgh, Edinburgh, United Kingdom; b Division of Infection and Immunity, The Roslin Institute, University of Edinburgh, Edinburgh, United Kingdom; c RIKEN Center for Life Sciences Technologies, Yokohama, Japan; d Clinical Biochemistry, Western General Hospital, Edinburgh, United Kingdom; e Mater Research Institute-University of Queensland, Brisbane, Australia; f Translational Research Institute, Brisbane, Australia; g Harry Perkins Institute of Medical Research, Nedlands, Australia; h RIKEN Preventive Medicine and Diagnosis Innovation Program, Wako, Japan; St. Jude Children's Research Hospital

**Keywords:** CAGE, NGS, cap-snatching, influenza, macrophages

## Abstract

Macrophages in the lung detect and respond to influenza A virus (IAV), determining the nature of the immune response. Using terminal-depth cap analysis of gene expression (CAGE), we quantified transcriptional activity of both host and pathogen over a 24-h time course of IAV infection in primary human monocyte-derived macrophages (MDMs). This method allowed us to observe heterogenous host sequences incorporated into IAV mRNA, “snatched” 5′ RNA caps, and corresponding RNA sequences from host RNAs. In order to determine whether cap-snatching is random or exhibits a bias, we systematically compared host sequences incorporated into viral mRNA (“snatched”) against a complete survey of all background host RNA in the same cells, at the same time. Using a computational strategy designed to eliminate sources of bias due to read length, sequencing depth, and multimapping, we were able to quantify overrepresentation of host RNA features among the sequences that were snatched by IAV. We demonstrate biased snatching of numerous host RNAs, particularly small nuclear RNAs (snRNAs), and avoidance of host transcripts encoding host ribosomal proteins, which are required by IAV for replication. We then used a systems approach to describe the transcriptional landscape of the host response to IAV, observing many new features, including a failure of IAV-treated MDMs to induce feedback inhibitors of inflammation, seen in response to other treatments.

**IMPORTANCE** Infection with influenza A virus (IAV) infection is responsible for an estimated 500,000 deaths and up to 5 million cases of severe respiratory illness each year. In this study, we looked at human primary immune cells (macrophages) infected with IAV. Our method allows us to look at both the host and the virus in parallel. We used these data to explore a process known as “cap-snatching,” where IAV snatches a short nucleotide sequence from capped host RNA. This process was believed to be random. We demonstrate biased snatching of numerous host RNAs, including those associated with snRNA transcription, and avoidance of host transcripts encoding host ribosomal proteins, which are required by IAV for replication. We then describe the transcriptional landscape of the host response to IAV, observing new features, including a failure of IAV-treated MDMs to induce feedback inhibitors of inflammation, seen in response to other treatments.

## Abstract (precis)

Infection with influenza A virus (IAV) infection is responsible for an estimated 500,000 deaths and up to 5 million cases of severe respiratory illness each year. In this study, we looked at human primary immune cells (macrophages) infected with IAV. Our method allows us to look at both the host and the virus in parallel. We used these data to explore a process known as “cap-snatching,” where IAV snatches a short nucleotide sequence from capped host RNA. This process was believed to be random. We demonstrate biased snatching of numerous host RNAs, including those associated with snRNA transcription, and avoidance of host transcripts encoding host ribosomal proteins, which are required by IAV for replication. We then describe the transcriptional landscape of the host response to IAV, observing new features, including a failure of IAV-treated MDMs to induce feedback inhibitors of inflammation, seen in response to other treatments.

## INTRODUCTION

Infection with influenza A virus (IAV) is responsible for an estimated 500,000 deaths and up to 5 million cases of severe respiratory illness each year (WHO) (1). The abundant macrophages of the airway and lung interstitium detect and respond to the virus, determining both the nature and the magnitude of the innate and acquired immune responses (2), and contribute to systemic inflammatory cytokine production in severe influenza (3).

As an obligate intracellular parasite, IAV is reliant on the host cellular machinery for replication. The IAV genome comprises 8 negative-sense RNA segments that are transcribed and replicated in the nucleus of the host cell. In order to coopt host translational machinery and to evade detection of non-self RNAs by host cells, IAV “snatches” 5′ RNA caps from host RNAs. The IAV polymerase binds directly to the 5′ 7-methylguanylate cap of a nascent host RNA and cleaves it roughly 10 to 14 nucleotides downstream. The snatched “leader” sequence is employed as a primer for efficient transcription of the viral mRNA (4), and subsequently, the host cap facilitates translation of viral mRNAs by host ribosomes. Previous large-scale studies of this process (5–8) have produced evidence that host-derived RNA caps are frequently snatched from noncoding RNAs, particularly small nuclear RNAs (snRNAs), due to their high abundance in infected cells. This has led to the conclusion that cap-snatching is not a selective process, that is, that capped host RNAs are snatched at random (8, 9).

Previous transcriptome sequencing studies have detected snatched leaders but have been unable to observe the complete pool of unsnatched sequences because of limited sequencing depth and resolution at the 5′ end, both of which are necessary to accurately quantify the background distribution of each host transcript.

To overcome these limitations, we utilized single-molecule terminal-depth cap analysis of gene expression (CAGE) to sequence all capped RNA from primary monocyte-derived macrophages (MDMs) from 4 human donors *in vitro* at 4 time points over the course of a 24-h productive infection with IAV. The CAGE RNA sequencing method captures both host- and virus-derived transcripts and, importantly, does not require a PCR amplification step, thus eliminating PCR bias.

By comparing the sequences of the snatched population to the sequences of the total capped RNA background, we observed biases in the snatching of transcripts encoding spliceosome components and avoidance of transcripts encoding host ribosomes.

This methodology allowed us to observe the transcriptional response to IAV infection over time in unprecedented molecular detail. We previously used CAGE to quantify transcript expression and promoter and enhancer activity in human MDMs and produced a detailed time course profiling their response to bacterial lipopolysaccharide (LPS) (10). In a comprehensive analysis of the host macrophage transcriptome during IAV exposure, we used a similar systems approach, using coexpression to identify key biological processes (11, 12) and compare the response of MDMs to both IAV and LPS, revealing IAV-specific features of the host response.

## RESULTS

### Transcriptional activity of IAV in human MDMs.

**FIG 1:**
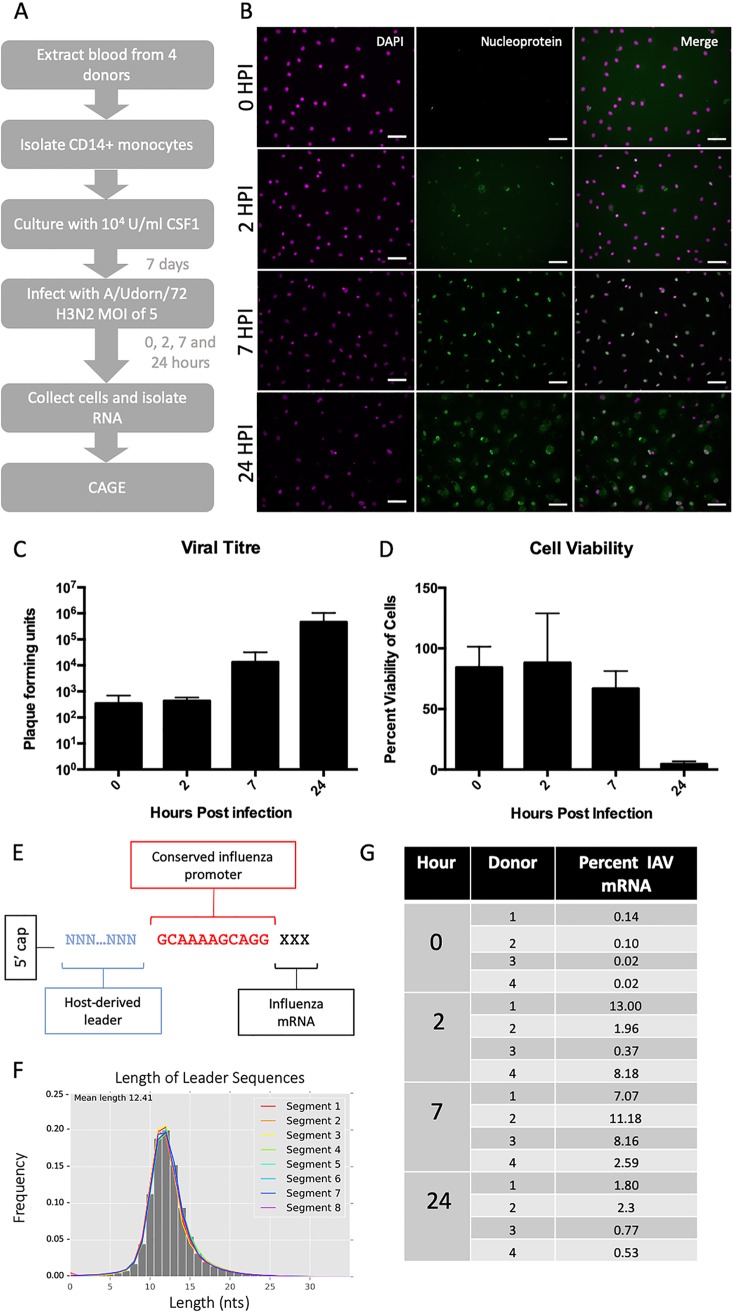
Characterization of human monocyte-derived macrophages productively infected with IAV. (A) Experimental outline. Blood was taken from 4 human donors, with appropriate ethical approval. CD14^+^ monocytes were extracted using magnetic beads and cultured in CSF1 for 8 to 10 days. MDMs were infected with A/Udorn/72 (H3N2) virus at a multiplicity of infection of 5. At 4 time points (0, 2, 7, and 24 h after medium change) the cells were collected and RNA isolated. (B) Human MDMs were stained using antibodies specific for viral nucleoprotein to confirm infection at 0, 2, 7, and 24 h postinfection. Scale bars, 10 μm. (C) Viral titer was measured by plaque assay at 0, 2, 7, and 24 h postinfection (*n* = 3 independent experiments) and shown as PFU/ml supernatant. (D) Cell viability was measured using Cell Titer Glo at 0, 2, 7, and 24 h postinfection (*n* = 3 independent experiments). (E) Schematic showing the structure of the capped 5′ end of IAV mRNAs. (F) Length of leader sequences across segments. (G) Frequency, as percentage, of IAV promoter-containing CAGE tags in each IAV-infected sample.

To observe IAV transcriptional dynamics in human MDMs *in vitro*, we infected MDMs from four different donors with influenza A/Udorn/72 (H3N2) virus (IAV) at a multiplicity of infection (MOI) of 5 (Fig. 1A). RNA libraries were prepared from cells at 0, 2, 7, and 24 h postinfection and from two uninfected infected samples at 0 and 24 h. Libraries were sequenced using HeliScope CAGE as previously described (11, 13). A minority of cells were positive for viral antigen (IAV nucleoprotein) by immunofluorescence after 2 h and the large majority were after 7 h (Fig. 1B), suggesting that viral mRNA molecules were being transcribed and translated. We confirmed a previous report (14) that IAV-infected MDM cells release infectious virus (Fig. 1C), albeit at approximately 10-fold-lower levels compared to published results for permissive cancer cell lines (15, 16) and with little evidence of cell death up to 7 h (Fig. 1D).

IAV mRNAs contain a conserved 12-base-long 5′-adjacent noncoding region (AGCAAAAGCAGG) present in all 8 segments derived from template-dependent transcription of the viral promoter (9). This sequence was used to identify viral transcripts. Similar to results seen elsewhere (6, 7, 17), the A at the 5′ end of the IAV promoter was not always present, and so sequences which contained the 11-nucleotide sequence GCAAAAGCAGG (IAV promoter) were brought forward for analysis (Fig. 1E).

Most (74%) of the leader sequences, those preceding the promoter, were between 10 and 14 nucleotides long (Fig. 1F). Published studies of IAV-infected A549 cells have reported that within 8 h postexposure, >50% of total cellular mRNA was viral (18). In contrast, IAV RNA constituted a relatively small proportion (4 to 11%) of total capped RNA in MDMs, even at the peak of viral replication (Fig. 1G).

**FIG 2:**
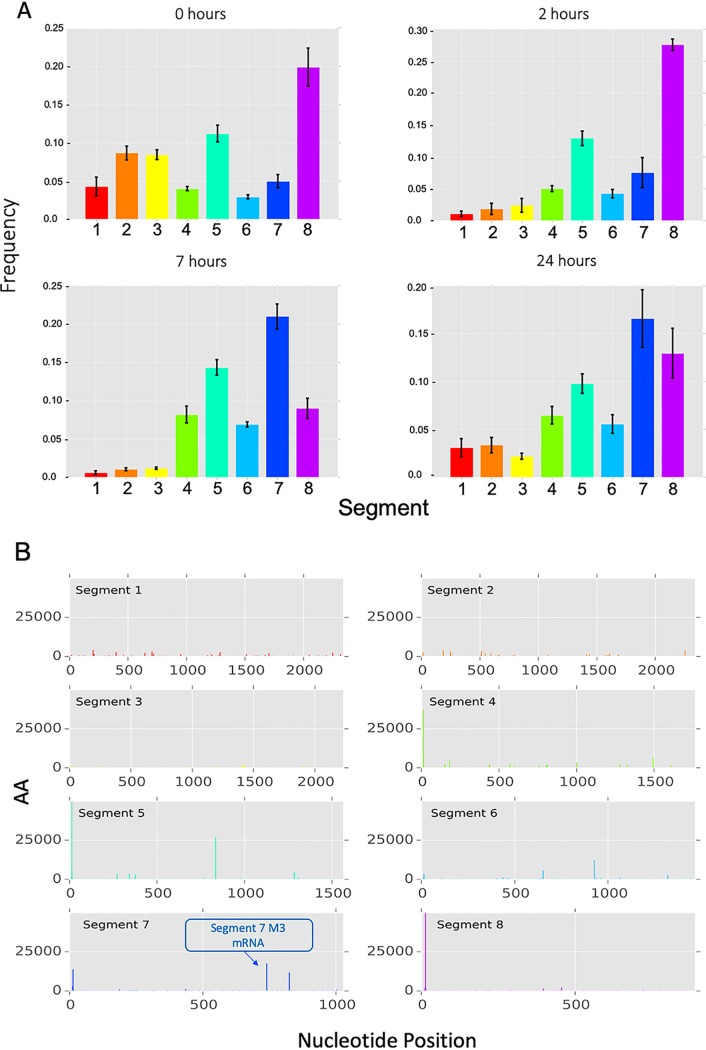
IAV segment transcriptional dynamics during infection of MDMs. (A) The relative amount, compared to the total amount of viral mRNA, of mRNA from each viral segment was calculated for individual donors at each of the four time points. Height of the bar represents the mean frequency between donors. Error bars show standard deviation. (B) The positions of potential splice variant sequences aligned to the Udorn genome are shown as adjusted abundance (AA). The known mRNA3 splice variant in segment 7 is shown (blue arrow). Time points and donors have been collated to increase signal.

The relative proportion of IAV mRNA arising from each viral segment was also consistent across the 4 donors at each time point (Fig. 2A), consistent with previous evidence that transcription of each segment is a highly controlled process (19). By 24 h, the pattern was less defined, which may be a consequence of mRNA decay and/or potential reinfection of the minor fraction of cells not infected at time zero.

### Potential alternative splice variants in IAV.

Splicing has been observed in segments 7 and 8 of IAV. In particular, segment 7 contains the splice donor site for the mRNA3/M3 transcript, which is found at the end of the promoter sequence (20). Over 400,000 reads contained the IAV promoter sequence and a leader sequence but did not originate from the genome sequence proximal to the promoter in any of the 8 segments. The leader and promoter sequences were removed and the sequences aligned throughout the Udorn genome. In order to quantify RNA expression at these loci, we summed the weighted abundances of reads originating at the same position. This revealed 6,902 putative capped IAV RNA sequences from the IAV genome, including the known splice variant of segment 7, the mRNA3 transcript (Fig. 2B). The alignments observed (see Table S1 in the supplemental material) are likely to include previously unidentified splice variants.

However, in a systematic search, no putative IAV splice variant RNA was preceded by a canonical major spliceosome acceptor site, apart from the mRNA3 transcript. It is possible that these represent variants that are expressed in such small amounts they are not detectable by other means, for example, Northern blotting or radioactive primer extension. It is of interest to determine if these putative mRNAs are true transcription products and if their transcription and translation contribute to viral pathogenesis.

### Characterization of host leader sequences incorporated into viral capped RNA.

We identified 4,575,918 unique leader sequences, heterogeneous in both sequence and length, snatched from the host and incorporated into viral mRNA. Contrary to previous reports (5, 8), we observed no difference in leader lengths between different viral segments. A total of 18.8% (859,789) of leader sequences appeared more than once and 1.5% (69,443) appeared ten times or more across all samples, indicating the presence of a highly snatched population.

We sought to determine whether there was overrepresentation of particular sequences, host transcripts, or biological pathways in the population of leader sequences compared to the background population of CAGE reads. In order to eliminate the risk of bias due to the different rates of successful mapping for sequences of different lengths, we restricted our analysis to the first 10 bases of every CAGE tag (10-mers), including both IAV and host sequences. The number of times a 10-mer was followed by the IAV promoter, i.e., incorporated into viral mRNA (“snatched”), was compared to the number of times a 10-mer was not followed by an IAV promoter (“unsnatched”), using Fisher’s exact test (false-discovery rate [FDR] of <0.05) at each time point. Of 29,195 10-mers meeting our minimum count threshold of 1,000 reads, we assigned a host transcript identity to 12,992 (44.5%). The remainder are a mixture of alternative host promoters, long noncoding RNAs (lncRNAs), enhancer RNAs (eRNAs), and other RNA species (21). Within these named 10-mers, 6,353 mapped ambiguously to more than one transcription initiation site, so a single identity was chosen at random from the possible sites. This approach decreased discovery power but was necessary to avoid bias that might be introduced into the identification based on a quantitative measure, such as abundance. The 1,000 most significantly enriched named genes in the snatched and unsnatched sets are reported in Table S2 in the supplemental material.

### Host snRNA is targeted by the cap-snatching mechanism.

**FIG 3:**
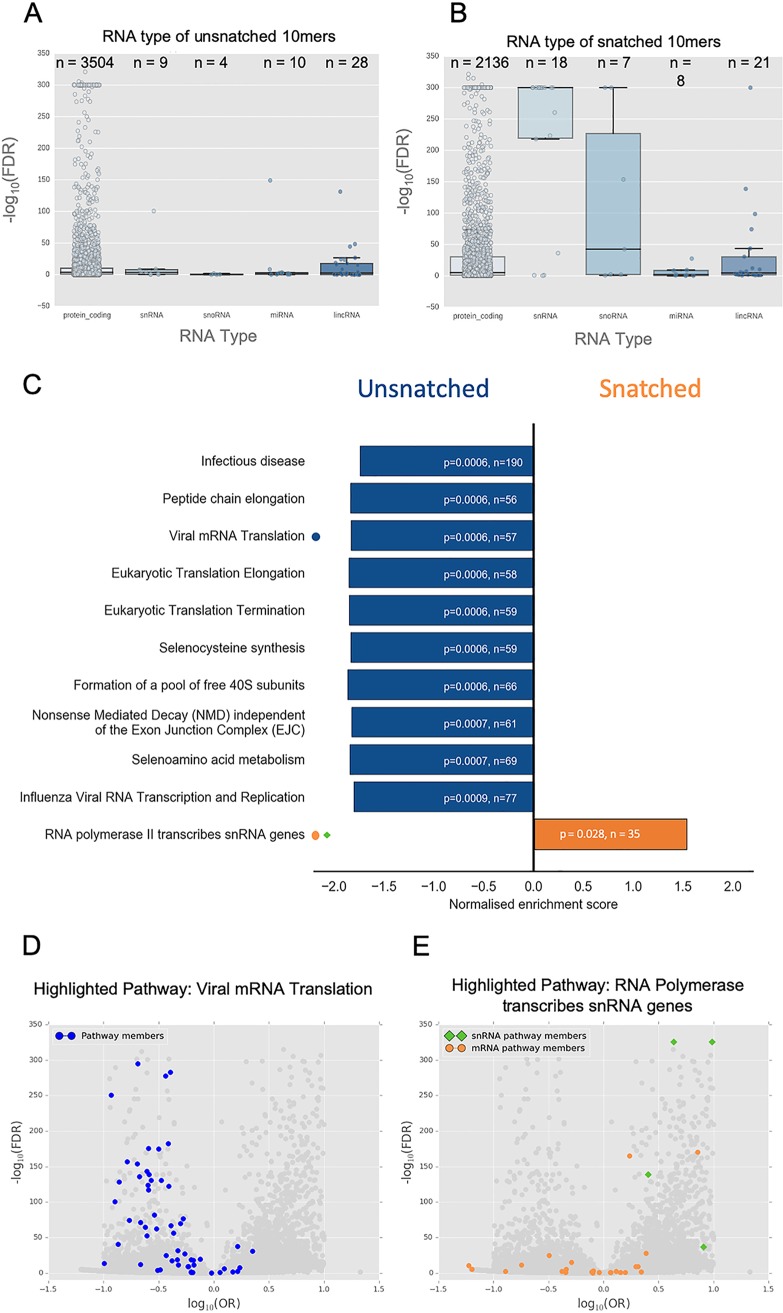
Pathway enrichment in snatched and unsnatched 10-mer sequences. (A and B) RNA type was assigned to 10-mers based on transcript identity. Only 10-mers with transcript identity were included. The significance of RNA type snatching was determined using analysis of variance (ANOVA). RNA types were plotted against –log_10_(FDR) for 10-mers of that type. The box denotes the interquartile range. The line within the box represents the average, and the whiskers represent the standard deviation. The individual data point for each 10-mer is also plotted. The number of 10-mers attributed to each RNA type is given as n above the box. (C) The 10 most underrepresented pathways (negative enrichment score, blue) and the single significantly overrepresented pathway (positive enrichment score, orange) in the Reactome 2016 database are shown. n represents the number of genes associated with that pathway detectable in the data set. *P* values shown are Benjamini-Hochberg FDR-adjusted *P* values. (D) Volcano plot showing the significance as −log_10_(FDR) and odds ratio of snatched versus unsnatched 10-mers with members of the Reactome pathway “RNA Polymerase transcribes snRNA genes” highlighted (snRNA, green diamonds; mRNA orange circles). (E) The same volcano plot as in panel D with members of the Reactome pathway “Viral mRNA Translation” highlighted (blue circles).

Key spliceosome snRNAs (RNU1, RNU11, RNU12, RNU4ATAC, RNU5A, RNU5E, RNU5F, RNU5D, and RNU7) and their variants/pseudogenes were among the most significantly enriched named genes. This is consistent with previous observations that snRNAs are snatched frequently (5, 6) and shows that this may represent a true preference for these RNAs. In view of this apparent preferential snatching of multiple snRNAs, we considered whether specific classes of capped host RNAs might be targeted. Of the RNA types considered, only snRNAs were strongly overrepresented in the snatched population (Fig. 3A and B). It is unclear at this time if both mature snRNAs (incorporated into the spliceosome) and immature snRNAs (prior to nuclear export and processing) are snatched as leader sequences.

This sequencing method also allows the observation of histone mRNA, which enabled us to observe that 10-mers corresponding to histone mRNAs were also significantly overrepresented.

The 10-mer corresponding to the transcript encoding the largest subunit of RNA polymerase II (*POLR2A*) was 5.83-fold overrepresented in snatched sequences (odds ratio [OR] = 5.83; FDR, <0.05). *PABPN1*, which encodes poly(A) binding protein, was also overrepresented (OR = 2.28; FDR, <0.05). These comprise key elements of both transcription and polyadenylation of host mRNAs.

Taken together, these observations might imply that cap-snatching interferes with regulation of transcription and splicing in the infected cell. However, *POLR2B*, encoding another subunit of RNA polymerase II, was 7.77-fold underrepresented (OR = 0.13; FDR, <0.05), making it difficult to draw simple conclusions from enrichment analysis that rely primarily on overlap statistics. To rectify this, we performed further gene set enrichment analyses that take into account background gene expression to determine statistically over- and underrepresented pathways affected by the cap-snatching mechanism.

### Specific ribosome-associated transcripts are avoided by the cap-snatching mechanism.

Identified transcripts from all time points and donors were collated and gene set enrichment analysis (with R package FGSEA) performed by querying various pathway/gene ontology data sets (listed in Table S3 in the supplemental material). Querying Reactome identified a single overrepresented pathway: RNA polymerase II transcribes snRNA genes (Fig. 3C). A volcano plot highlighting the distribution of pathway members shows that many mRNA pathway members were underrepresented and that its enrichment as an overrepresented pathway was driven by snRNA transcripts (Fig. 3E), particularly snRNA members of the minor spliceosome. This is consistent with the observed apparent preferential snatching of snRNAs.

Pathway enrichment also allowed us to look for pathways that were avoided by the cap-snatching mechanism. We identified pathways associated with translation and ribosome formation as significantly underrepresented in the cap-snatched pool (Fig. 3D). Although multiple pathways were identified, these were not independent; these associations were largely driven by the presence of a group of transcripts encoding the same set of ribosomal proteins (Table S3). These data show that IAV avoids snatching caps from ribosomal mRNA transcripts. Interestingly, not all mRNAs encoding ribosomal subunits were avoided. We compared our results to a recent study reporting the effect of targeted knockdown of specific ribosomal subunit mRNAs in the context of IAV infection (22), but we saw no clear relationship between cap-snatching preference and viral protein production, host protein production, or antigen presentation.

### Enrichment of specific RNA motifs in the snatched and unsnatched sequence populations.

Leader sequences are known to commonly have GCA at the interface between the host sequence and the IAV promoter (23, 24) introduced partially through the “prime-and-realign” mechanism (17, 24, 25). More recently, an AG at the 5′ end of the leader sequence has also been shown to be prevalent in snatched sequences (6).

**FIG 4:**
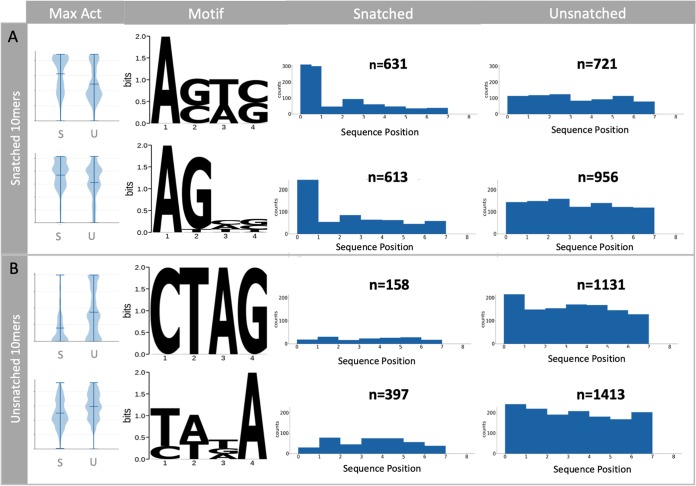
Nucleotide motifs associated with snatched and unsnatched 10-mer sequences. The first 10 nucleotides of each CAGE tag were extracted, and the abundance of each sequence associated with IAV was compared to the background abundance by Fisher’s exact test (FDR < 0.05). Identification of motifs associated with snatched (A) and unsnatched (B) sequences is shown. Violin plots show the maximum activation distributions for snatched (S) and unsnatched (U) sequence categories in arbitrary units. The four-nucleotide-long motifs associated with each category are visualized as position weight matrices. The positional enrichment of the four-nucleotide motifs across the 10-mer sequences is shown. The number of sequences is given as n above each bar chart.

Our analysis of 10-mers enables a statistically powerful comparison of snatched and unsnatched sequences in which the positions of sequence motifs can be compared without reference to distance from the 5′ or 3′ ends. We used Pysster (26) to train convolutional neural networks using the sequence data to explore sequence and positional features, of a length of 4 nucleotides, for pools of highly significantly overrepresented snatched and unsnatched 10-mers [0.3 ≥ OR ≥ 3, −log(FDR) < 10]. This stringency was introduced to eliminate potential noise. The snatched 10-mers showed an enrichment of two motifs, A[G/C][T/A][C/G] and the similar sequence AGNN, both beginning at the first base (position 0) (Fig. 4A). These motifs were most apparent at 2 h postinfection, coinciding with levels of high transcription by the virus, and are consistent with previous reports of an AG preference at the 5′ end of the leader (6).

The unsnatched 10-mers also showed an enrichment of two distinct motifs, CTAG and [T/C][A/T][T/G/A]A, most evident at 7 h postinfection (Fig. 4B). While the CTAG motif was unsnatched primarily when it began in the first position (position 0), there was also an association between this motif at any position in the 10-mer and unsnatched status. Similarly, the [T/C][A/T][T/G/A]A motif was avoided by cap snatching if it occurred at any position within the 10-mer (Fig. 4B). To our knowledge this is the first evidence for the avoidance of particular sequences as priming leaders by the IAV polymerase.

### Network analysis of the response to IAV infection in MDMs.

Temporal changes in host cell transcription are likely to occur both in recognition of viral infection and as a consequence of viral life cycle progression. IAV can dysregulate host transcription, in a manner which leaves transcription initiation apparently unaffected (27). The advantage of CAGE in this scenario is the snapshot of transcription initiation it provides, in contrast to other techniques, such as RNA-seq, which sequence the entire mRNA molecule, including downstream-of-gene transcripts (28).

We utilized the network analytical tool Graphia (29) to identify sets of coregulated transcripts in the MDM response to IAV (see Table S4 in the supplemental material). For simplicity, we restricted the analysis to the dominant (most frequently used) promoters (p1) and used averaged data from the 4 donors. We have summarized the Gene Ontology (GO) term enrichment and pathway enrichment in the 10 largest clusters using GATHER (30) (see Table S5 in the supplemental material) and Enrichr (31, 32) (see Table S6 in the supplemental material), respectively.

**FIG 5:**
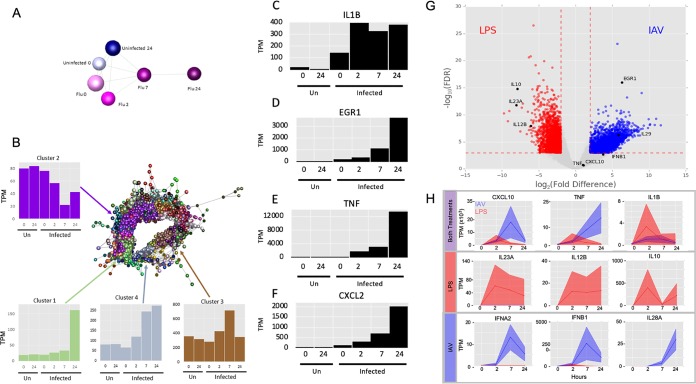
Network analysis of the coexpressed genes during IAV infection in MDMs. (A) Sample-to-sample network. A correlation coefficient of ≥0.7 was used to include all samples in the network. Analysis was restricted to the dominant promoters (p1), and data were averaged across the 4 donors. Blue, uninfected; pink, infected. Darker colors show later time points. (B) Gene-to-gene correlation profile of transcripts. Network analysis identified the sets of coregulated transcripts in the MDM response to IAV. Analysis was restricted to the dominant promoters (p1), and data were averaged across the 4 donors. Lines represent connections at a Pearson correlation coefficient of ≥0.94, and spheres represent genes (promoters). The clustering procedure used a relatively coarse Markov clustering algorithm of 1.7 to avoid excessive cluster fragmentation. The four largest clusters, along with their average expression profiles, are shown. The *y* axis in the expression profiles shows the expression level in tags per million (TPM). (C to F) Abundance of transcripts for IL-1β (C), EGR1 (D), TNF-α (E), and CXCL2 (F) at the indicated time points. The *y* axis shows expression in tags per million (TPM). (G) Differential gene expression analysis comparing expression of transcripts in LPS- treated and IAV-treated MDMs. Transcripts with a relative log_2_ fold change of ≥2 and a −log_10_(FDR) of ≥3 are shown in red (higher with LPS treatment) and blue (higher with IAV infection). Genes with the greatest difference in expression are labeled. Genes referenced in the text are shown in black. (H) Comparison of the temporal response of genes between IAV- and LPS-treated MDMs. Expression (TPM) of selected genes in LPS-treated (red) and IAV-infected (blue) human MDMs at 0, 2, 7, and 24 h posttreatment is shown in tags per million (TPM). Solid lines show the mean expression of all donors (*n* = 3 for LPS and *n* = 4 for IAV). Filled-in areas show standard deviation between donors.

Figure 5A shows the sample-to-sample correlation graph for each of the averaged data sets. Although there was a global alteration in transcript induction that progressed with time, the profile at 7 h remained correlated with the profiles in uninfected cells at both early and late time points. This suggests that the virus did not cause a selective, or global, loss of host transcription initiation. In keeping with that conclusion, the largest cluster, cluster 1, contained more than 4,500 genes (Fig. 5B) whose shared pattern was continuous induction across the time course with particularly high initiation at 24 h. This cluster contained genes encoding the interferon-responsive transcription factors, *IRF1*, -*2*, -*4*, -*7*, -*8*, and -*9* and numerous known interferon-responsive antiviral effector genes (e.g., *APOBEC3G*, *RSAD2*, *DDX58*, *ISG15*, *MX1*, *OAS1*, and *TRIM25*).

We observed that the response of MDMs to viral infection was immediate. Interleukin 1β (IL-1β) was rapidly and strongly induced by IAV at 0 h (effectively 1 h after virus addition) and peaked at 2 h (3 h after virus addition) (Fig. 5C). Other early response genes that were detected early after IAV exposure included those encoding immediate early transcription factors such as EGR1, the proinflammatory cytokine tumor necrosis factor alpha (TNF-α), and the neutrophil chemoattractant CXCL2 (Fig. 5D to F).

Rapidly induced genes are concentrated in clusters 3 and 4, including interferon (IFN) genes *IFNB1*, *IFNA1*, *IFNA2*, *IFNA8*, *IFNA14*, and *IFNE* and further known IFN-regulated targets such as *IFI6*, *IFIT2*, *IFITM3*, *IRG1*, *GBP1*, and *MNDA*. Also enriched in these clusters are genes involved in protein synthesis, including 46 ribosomal protein subunit genes, which are avoided by IAV cap-snatching (see above).

### Comparative analysis of the response of MDMs to treatment with IAV and with LPS.

**FIG 6:**
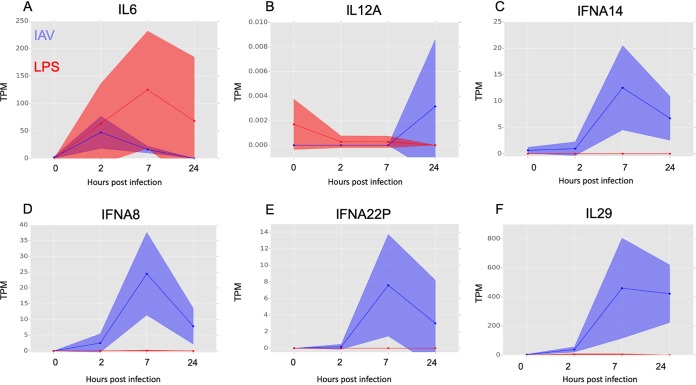
Comparative analysis of the response of MDMs to treatment with IAV and with LPS. The relative expression of selected genes in LPS-treated (red) and IAV-infected (blue) human MDMs at 0, 2, 7, and 24 h posttreatment is shown in tags per million (TPM). Solid lines show the mean expression of all donors, and filled-in areas show standard deviation between donors (*n* = 3 for LPS and *n* = 4 for IAV).

The responses of MDMs to IAV and LPS were compared at equivalent time points, uncovering some common transcripts that were induced in both treatments (Fig. 5H, top row). Transcripts induced specifically by LPS but not by IAV were revealed by differential expression analysis (Fig. 5G; see Table S7 in the supplemental material) and included classical inflammatory cytokines IL-12β (although not IL-12α) and IL-6 and the feedback regulator of inflammation, IL-10 (Fig. 5H [central row] and Fig. 6A and B). Conversely, induction of genes associated with interferon signaling was more substantial and prolonged in IAV-treated MDMs than those treated with LPS. IAV induced *IFNB1* mRNA ca. 10-fold more than observed in response to LPS in MDMs and sustained this expression throughout the time course (Fig. 5H, bottom row). IAV also induced multiple IFNA genes (*IFNA1*, -*A2*, -*A8*, -*A14*, and -*A22*) (Fig. 6C to E) and the type III interferon genes *IFNL1* (*IL28A*) and *IFNL2* (*IL-29*), which were not induced at all by LPS (Fig. 5H [bottom row] and Fig. 6F).

## DISCUSSION

This comprehensive analysis of host and viral transcripts reveals key features of the host-pathogen interaction at a molecular level. We demonstrate that IAV cap-snatching is biased toward host transcripts associated with splicing and avoids host ribosomal subunit transcripts. Additionally, we provide a comprehensive analysis of transcripts initiated as part of the host response to IAV in a vital innate immune cell.

### Elimination of bias for accurate quantification of leader sequences and 5′ RNA ends.

Our choice of sequencing methodology and analytical approach eliminated numerous sources of bias that have limited the interpretation of previous studies of cap-snatching preference.

The HeliScope single-molecule CAGE sequencing methodology sequences transcripts from the 5′ end without internal segment-specific primers and without PCR amplification (13). In contrast, previous studies of IAV transcripts used internal primers for the viral segments (5, 8) or performed library amplification on cDNA derived from capped RNA (6). A key difference from previous work is the quantification of background transcription, which enables the first accurate quantification of the transcripts not snatched by IAV.

In addition, our use of terminal-depth sequencing limits noise and sampling error, in both the snatched sequences and the background distribution. Since CAGE reads sequences directly from the 5′ end, we can be confident that we have quantified the background pool of potential leader sequences that were available to be snatched. By limiting our analysis to sequences of a specific length (10-mers), we eliminate bias that may occur due to differential mapping or identification of sequences of different lengths.

### The cap-snatching mechanism is not entirely random.

Although random cap-snatching does occur, 18.8% of leaders are snatched multiple times, and our analysis shows that many are snatched more frequently than one would expect from the level of background RNA expression. Noncoding RNAs, particularly snRNAs, have been identified as the source of the most frequently snatched leader sequences (5, 6). However, it was unclear whether this frequency reflected the high abundance of these transcripts in infected cells or a true overrepresentation of this RNA type among leaders. Our analysis enables an unbiased, accurate quantification of the abundance of each sequence in both the snatched and unsnatched sequence sets.

Differential expression analysis revealed that all snRNAs, apart from RNU1, were upregulated in IAV-treated MDMs compared to LPS (see Table S7 in the supplemental material). Notably, snRNA components of the minor spliceosome, (*RNU11*, *RNU12*, *RNU4ATAC*, *RNU5A*, and *RNU5E*), a molecular machine that splices <1% of introns in the human genome, were highly snatched compared to background expression, particularly at 2 and 7 h.

If cap-snatching were determined only by abundance, as previously thought (8, 9), we would expect to see leader sequences derived from ribosomal genes prominently among the snatched sequences. Our comparison of LPS- and IAV-treated cells shows that genes encoding ribosomal subunits are highly transcribed in IAV-treated cells. Although we do see a minority of mRNAs encoding ribosomal proteins in the snatched set, IAV cap-snatching exhibited a surprisingly strong avoidance of most mRNAs encoding ribosomal proteins, which is particularly evident in pathway enrichment analysis.

### Characteristics of MDM response to IAV.

The snapshot of transcription initiation provided by CAGE analysis allowed us to examine coexpression clusters and observe the consistent similarity in global transcription initiation between uninfected and early-postinfection MDMs, suggesting that most basic cellular processes are maintained during infection in this model. The largest coexpression cluster, cluster 1 (see Table S4 in the supplemental material), included genes encoding the ubiquitin-proteasome complex, oxidative phosphorylation, and cell cycle and transcriptional regulation, including mRNA splicing and binding. In A549 cells, IAV infection causes cell cycle arrest (33) and downregulation of cell cycle-associated genes. Since MDMs are not actively proliferative, the apparent induction by IAV infection of many cell cycle-related genes, including cyclin genes and 19 genes encoding multiple cyclin-dependent kinases (CDKs), is unlikely to be associated with cellular proliferation.

### Comparison between the host responses in IAV- and LPS-treated MDMs.

Like LPS, IAV strongly induced TNF-α, IL-1β, multiple chemokine genes (e.g., *CCL2*, *CCL3*, *CXCL1*, *CXCL2*, and *CCL20*), and many genes for immediate early transcription factors (e.g., the *EGR* family). However, the global transcript initiation-based analysis of the response to IAV reveals a clear contrast to the LPS response in MDMs. In LPS treatment, levels of many inflammatory transcripts are subject to control by a complex network of rapidly induced feedback regulators (10). The sustained induction of proinflammatory transcripts in response to IAV contrasts with this transient induction in response to LPS.

Following LPS treatment, MDMs have low initiation of *IL12A* (p35) mRNA, instead inducing *IL-23A* and *IL12B* mRNAs, which together encode the heterodimeric proinflammatory cytokine IL-23. These were not detected in IAV-infected cells. Similarly, there was no detectable induction of the anti-inflammatory cytokine *IL-10* mRNA by IAV, while transcript initiation was massive and sustained in LPS-treated cells.

The type III interferons were specific to IAV-treated MDMs. These were recently shown to mediate a key mechanism preventing viral spread to the lower respiratory tract in mice (34), which is believed to cause life-threatening disease in humans (35). The profound difference in induction of IFN-responsive genes in this cell type between LPS and IAV stimulation is reflective of blood transcriptome profiles of patients with severe IAV compared to those with bacterial sepsis (36).

### Limitations of this study.

Our study is, to our knowledge, the most comprehensive systems-level evaluation of both host and viral transcriptional activity for IAV replication and the first study to perform an unbiased quantification of cap-snatching preference compared with accurate measurement of background transcription. It is, however, limited to a single cell type and one strain of IAV. It is possible that the observed apparent preference for and avoidance of specific capped RNA are specific to MDMs. The observation that snRNAs RNU1 and RNU2 are the most frequently snatched sequences in H1N1-infected A549 cells in other studies (6, 17) indicates that it is reasonable to speculate that this mechanism is generalizable across other types. Finally, our method measures total capped RNA and does not differentiate between nuclear and cytoplasmic RNA molecules.

Future work is needed to explore the mechanisms underlying the preference for and avoidance of specific mRNAs and to determine cap-snatching preferences of other IAV strains.

## MATERIALS AND METHODS

### Ethics, cell culture, virus propagation, and infections.

Cells were isolated from fresh blood from volunteer donors under ethical approval from the Lothian Research Ethics Committee (11/AL/0168). Primary CD14^+^ human monocytes were isolated from whole blood from 4 human donors as described previously (37). Monocytes were plated for 7 days in RPMI 1640 supplemented with 10% (vol/vol) fetal bovine serum (FBS), 2 mM glutamine, 100 U/ml penicillin, 100 μg/ml streptomycin (Sigma Co.), and 104 U/ml (100 ng/ml) recombinant human colony-stimulating factor 1 (rhCSF1) (a gift from Chiron, Emeryville, CA, USA) for differentiation into macrophages. Cells were maintained at 37°C with 5% CO_2_. A/Udorn/72 (H3N2) virus was generated as described previously (14). Differentiated macrophages were infected on day 8. Cells were washed in serum-free medium, after which they were infected at an MOI of 5 in a volume of 200 μl infection medium. Cells were incubated for 1 h at 37°C and then washed three times with serum-free medium and incubated in RPMI 1640 supplemented with 1 μg/ml tosylsulfonyl phenylalanyl chloromethyl ketone (TPCK)-trypsin, 0.7% bovine serum albumin (BSA), 2 mM glutamine, 100 U/ml penicillin, 100 μg/ml streptomycin (Sigma Co.), and 104 U/ml (100 ng/ml) rhCSF1. Samples were collected at 4 time points after infection/medium change: 0 h (1 h after addition of the virus), 2 h, 7 h, and 24 h. Uninfected samples were also collected at 0 and 24 h. LPS treatments were carried out as described previously (10). Only time points with corresponding IAV treatment time points were used in this analysis.

### Immunofluorescence.

Primary human monocyte-derived macrophages were differentiated, as described above, on glass coverslips. Cells were infected as described above. At 0, 2, 7, and 24 h postinfection, cells were fixed for 20 min in 4% formaldehyde in phosphate-buffered saline (PBS). After permeabilization with 0.2% Triton X-100 in PBS for 5 min at room temperature, cells were incubated with mouse monoclonal influenza A NP AA5H (Bio-Rad) at 1:500. After 1 h, cells were washed three times with PBS and incubated with goat anti-mouse Alexa Fluor 488 at 1:1,000 (Thermo Fisher). After 1 h, cells were washed three times with PBS and incubated in 4′,6′-diamidino-2-phenylindole (DAPI) (Thermo Fisher) for 10 minutes, after which they were washed three times with PBS and mounted on slides using Vectashield Antifade mounting medium. Cells were viewed on a Leica fluorescence upright microscope and imaged using a Hamamatsu Orca-ER low-light monocamera. Scale bars were added using ImageJ.

### Cell viability and virus titration.

Cell viability was measured using Cell Titer Glo at 0, 2, 7, and 24 h postinfection. Virus produced was titrated by plaque assay on MDCK cells. Virus titers in cell supernatants were determined by plaque titration using 10-fold serial dilutions of virus stocks. Confluent MDCK cells in 6-well plates were inoculated with cell supernatant for 1 h in serum-free medium. An overlay (a mixture of equal volumes of Dulbecco modified Eagle medium [DMEM] and 2.4% Avicel [Sigma-Aldrich, UK] supplemented with 1 μg/ml TPCK-treated trypsin and 0.14% BSA fraction V) was then put onto the wells. After 48 h, cells were fixed using 3.5% formaldehyde and stained with 0.1% crystal violet. Virus titers were calculated by plaque count × dilution factor/volume of inoculum and expressed as PFU per milliliter of supernatant.

### CAGE.

RNA was extracted using the Qiagen miRNeasy minikit (217004). RNA quality was assessed and CAGE was performed as described previously (38) as part of the FANTOM5 project. Virus genome information is available in Table S8 in the supplemental material.

### Data analysis and identification of IAV mRNA.

Computational analysis was performed using custom Python scripts and as described previously (11). Capped IAV RNAs were identified by the conserved 11-base promoter sequence expected to be in all viral mRNA (GCAAAAGCAGG), as described in Results. Sequences that contained the promoter were classified as capped viral mRNA and aligned to the Udorn sequence.

### Unbiased analysis of leader sequence preference.

The first 10 nucleotides of each CAGE tag (10-mers) that reached the abundance threshold in our data set were extracted, and this set of unique 10-mers was used in subsequent analysis. The abundance threshold was set to 1,000 occurrences across all samples. To determine the 10-mer sequences that were over- and underrepresented in the snatched population based on background abundance, the number of times a 10-mer was associated with the IAV promoter (“snatched”) was counted along with the number of times the 10-mer occurred without the promoter (“unsnatched”). These were analyzed using Fisher’s exact test. The Benjamini-Hochberg correction was applied to *P* values. Significance was determined by an FDR of <0.05. The number of times a 10-mer was snatched was compared to the number of times it occurred unsnatched at the previous time point by Fisher’s exact test.

### Assignment of transcript identity to 10-mer sequences.

CAGE tags were mapped to the human reference genome (hg19) as described previously (11). We extracted every possible chromosomal location for a 10-mer that met the abundance threshold of 1,000 across all samples from the original alignment BAMfiles created as part of the FANTOM5 project. Ten-mers containing a 6-mer from within the IAV promoter (GCAAAA, CAAAAG, AAAAGC, AAAGCA, AAGCAG, and AGCAGG) were removed. Reference transcription start sites were downloaded from FANTOM5. Promoter identity was assigned first using BEDtools 2.25.0 with a window of ±5 bases and exact strand match only. For each possible promoter identity, the 10-mer sequence was mapped to the genomic sequence with a window of ±5 bases directly, and exact matches only were used to assign promoter identity.

A promoter identity was chosen at random from the list of mapped sites to avoid any effect of abundance that may bias transcript identification. Promoter identities were converted to HGNC format. To determine preference of promoters and genes in leader sequences, all 10-mers that were assigned to that promoter or gene name were counted and Fisher’s exact test was performed. Benjamini-Hochberg FDRs were calculated using the scipy.stats v 0.18.1 statsmodels.stats.multitest.mutlipletests function with method = ‘fdr_bh’. Significance was determined by an FDR of <0.05. RNA type was assigned to named transcripts using reference data downloaded from Biomart (http://www.ensembl.org).

### Pathway and gene set enrichment analysis.

GO term assignment and pathway analysis for coexpression clusters were performed using Enrichr (amp.pharm.mssm.edu/Enrichr) (31, 32) and GATHER (30). Pathway databases queried were Reactome 2016, KEGG 2016, WikiPathways 2016, GO Molecular Function 2015, GO Cellular Component 2015, and GO Biological Process 2015. Gene set enrichment analysis on ranked cap-snatching preference data was performed using R package FGSEA (39) in R version 3.5.1 with the following parameters: set.seed = 42, min set size = 5, max size = 5000, nproc = 1, and nperm = 1000000. Gene set libraries KEGG 2016, BioCarta 2016, Reactome 2016, WikiPathways 2016, NCI Nature 2016, GO Biological Process 2018, GO Molecular Function 2018, and GO Cellular Component 2018 were used. Genes were ranked by −log_10_(*P* value) and log_10_(OR). The Benjamini-Hochberg correction was applied to *P* values. All named genes that appeared significant were included in this analysis.

### Analysis of leader motifs using convolutional neural networks.

A subset of 10-mers that reached the following thresholds of 0.3 < OR > 3 and −log(FDR) < 10 were brought forward for analysis of motif preference using convolutional neural networks. We optimized an existing network (26) for our use by using altering the parameters to find suitable settings. Optimization experiments demonstrated that a kernel length of 4 gave us relatively high and relatively consistent precision and recall by using the grid search to explore various kernel lengths (2, 3, 4, and 5) and drop rates (0, 0.1, and 0.5); for other parameters, we used the default settings of Pysster (kernel number, 20; convolutional layer number, 2) apart from learning rate at 0.0001 and patience, stopping at 100. Since our analysis was restricted to 10-mers, we did not use the pooling method. We randomly selected the training set and validation set in the proportion of 60% and 30% independently. Motifs were considered if they reached a score of at least 50% of the maximum score for that time point.

### Identification of potential alternative splice variants.

CAGE tags containing a leader sequence and an IAV promoter sequence followed by a sequence that did not align proximal to the IAV promoter sequence in the Udorn genome were extracted. These novel “promoter-proximal” sequences were hypothesized to be derived from putative 5′ untranslated region (5′UTR) sequences internal to a segment arising from mRNA from splice variants. These sequences were aligned throughout the Udorn genome using custom Python scripts. The abundance of each sequence was divided by the number of locations in the Udorn genome to which it could map. The weighted abundances at each position were then summed and graphed. Segment 7 mRNA3 was used as a proof of principle

### Network analysis of the MDM transcriptome during infection.

Network analysis of the MDM transcriptome during infection was carried out using Graphia Professional (Kajeka Ltd., United Kingdom [formerly Biolayout Express3D]). Results were filtered to exclude any transcript where the maximum value across all samples did not reach 10 tags per million (TPM). The sample-to-sample analysis was performed at a Pearson correlation coefficient of ≥0.70. The gene-to-gene analysis was performed at a Pearson correlation coefficient of ≥0.94 and used a relatively coarse Markov cluster algorithm inflation value of 1.7 to avoid excessive cluster fragmentation. We restricted the analysis to the dominant promoters (p1) and used averaged data from the 4 donors.

### EdgeR analysis of LPS-treated versus IAV-treated samples.

Differential expression between groups of genes was analyzed using the EdgeR package (40) in R version 3.5.1. CAGE data for LPS and IAV data sets were processed as described previously (10). Clustered transcription start sites (CTSS) with a minimum expression level of 10 tags per million in at least one comparable time point and with a coefficient of variation of >0.5 were included in expression analysis. Samples corresponding to 7 h posttreatment were carried forward for analysis. We used the glmFit function to fit the models and glmLRT to perform testing between the LPS- and IAV-treated samples. The Benjamini-Hochberg correction was applied to *P* values. A significance threshold of FDR < 0.05 was used.

### Data availability.

Custom Python scripts are available at https://github.com/baillielab/influenza_cage. CAGE data are available to download at http://fantom.gsc.riken.jp/5/data/.

## Supplementary Material

Supplemental file 1

Supplemental file 2

Supplemental file 3

Supplemental file 4

Supplemental file 5

Supplemental file 6

Supplemental file 7
