# Supplementary material for: Comprehensive Characterization of Transcriptional Activity during Influenza A Virus Infection Reveals Biases in Cap-Snatching of Host RNA Sequences
Source: J Virol. 2020 May 4;94(10):e01720-19. doi: 10.1128/JVI.01720-19 (PMC7199409; doi:10.1128/JVI.01720-19)
Supplement: Supplemental file 1 [file JVI.01720-19-s0001.pdf]

Table S1: mRNA sequences for A/Udorn/H3N2 putative splice variants.

>udornh3n2_segment7_825_nt
AGCAAAAGCAGGTGATATTGTGGATTCTTGATCGTCTTTTTTTCAAATGCATCTATCGATTCTTTGAA
CACGGTCTGAAAAGAGGGCCTTCTACGGAAGGAGTACCTGAGTCTATGAGGGAAGAATATCGAAAGGA
ACAGCAGAGTGCTGTGGATGCTGACGACAGTCATTTTGTCAGCATAGAGCTGGAGTAAAAAACTACCT
TGTTTCTACT
>udornh3n2_segment6_926_nt
AGCAAAAGCAGGTAGATATAAATGTGAAAGATTATAGCATTGATTCCAGTTATGTGTGCTCAGGGCTT
GTTGGCGACACACCCAGAAACAACGACAGATCTAGCAATAGCTATTGCCGGAATCCTAACAATGAGAA
AGGGAATCACGGAGTGAAAGGCTGGGCCTTTGACGATGGAAATGACGTGTGGATGGGAAGAACGATCA
GCGAGGATTCACGCTCAGGTTATGAAACCTTCAAAGTCATTGGTGGTTGGTCCACACCTAATTCCAAA
TTGCAGATAAATAGGCAAGTCATAGTTGACAGCGATAATAGGTCAGGTTATTCTGGTATTTTCTCTGT
TGAGGGCAAAAGCTGCATCAATAGGTGCTTTTATGTGGAGTTGATAAGGGGAAGGGAACAGGAAACTA
GAGTATGGTGGACCTCAAACAGTATTGTTGTGTTTTGTGGCACTTCAGGTACCTATGGGACAGGCTCA
TGGCCTGATGGGGCGGACATCAATCTCATGCCTATATAAGCTTTCGCAATTTTAGAAAAAAACTCCTT
GTTTCTACT
>udornh3n2_segment6_652_nt
AGCAAAAGCAGGGTAGACAGTATTGGTTCATGGTCTCAAAATATCCTCAGGACCCAGGAGTCGGAATG
CGTTTGTATCAATGGGACTTGTACAGTAGTAATGACTGATGGAAGTGCTTCAGGAAGAGCTGATACTA
AAATACTATTCATTGAAGAGGGGAAAATTGTTCATATTAGCCCATTGTCAGGAAGTGCTCAGCATGTA
GAGGAGTGTTCCTGTTATCCTCGATATCCTGGTGTCAGATGTATCTGCAGAGACAACTGGAAAGGCTC
TAATAGGCCCGTCGTAGATATAAATGTGAAAGATTATAGCATTGATTCCAGTTATGTGTGCTCAGGGC
TTGTTGGCGACACACCCAGAAACAACGACAGATCTAGCAATAGCTATTGCCGGAATCCTAACAATGAG
AAAGGGAATCACGGAGTGAAAGGCTGGGCCTTTGACGATGGAAATGACGTGTGGATGGGAAGAACGAT
CAGCGAGGATTCACGCTCAGGTTATGAAACCTTCAAAGTCATTGGTGGTTGGTCCACACCTAATTCCA
AATTGCAGATAAATAGGCAAGTCATAGTTGACAGCGATAATAGGTCAGGTTATTCTGGTATTTTCTCT
GTTGAGGGCAAAAGCTGCATCAATAGGTGCTTTTATGTGGAGTTGATAAGGGGAAGGGAACAGGAAAC
TAGAGTATGGTGGACCTCAAACAGTATTGTTGTGTTTTGTGGCACTTCAGGTACCTATGGGACAGGCT
CATGGCCTGATGGGGCGGACATCAATCTCATGCCTATATAAGCTTTCGCAATTTTAGAAAAAAACTCC
TTGTTTCTACT
>udornh3n2_segment5_835_nt
AGCAAAAGCAGGTAATATTGAGAGGGTCAGTTGCTCACAAATCTTGTCTGCCTGCCTGTGTGTATGGA
CCTGCCGTAGCCAGTGGGTACGACTTCGAAAAAGAGGGATACTCTTTAGTGGGAATAGACCCTTTCAA
ACTACTTCAAAACAGCCAAGTATACAGCCTAATCAGACCGAACGAGAATCCAGCACACAAGAGTCAGC
TGGTGTGGATGGCATGCAATTCTGCTGCATTTGAAGATCTAAGATTATTAAGCTTCATCAGAGGGACC
AAAGTATCTCCAAGGGGGAAACTTTCAACTAGAGGAGTACAAATTGCTTCAAATGAAAACATGGATAC
TATGGAATCAAGTACTCTTGAACTGAGAAGCAGGTACTGGGCCATAAGGACCAGAAGTGGAGGAAACA
CTAATCAACAGAGGGCCTCCGCAGGCCAAATCAGTGTGCAACCTGCATTTTCTGTGCAAAGAAACCTC
CCATTTGACAAATCAACCATCATGGCAGCATTCACTGGGAATACGGAGGGAAGAACCTCAGACATGAG
GGCAGAAATCATAAGGATGATGGAAGGTGCAAAACCAGAAGAAGTGTCCTTCCGTGGGCGGGGAGTCT
TCGAGCTCTCGGACGAAAAGGCAACGAACCCGATCGTGCCCTCTTTTGACATGAGTAATGAAGGATCT
TATTTCTTCGGAGACAATGCAGAGGAGTACGACAATTAAGGAAAAATACCCTTGTTTCTACT
>udornh3n2_segment4_1493_nt
AGCAAAAGCAGGTGTGACAATGCTTGCATAGGGTCAATCAGAAATGGGACTTATGACCATGATGTATA
CAGAGACGAAGCATTAAACAACCGGTTTCAGATCAAAGGTGTTGAACTGAAGTCAGGATACAAAGACT
GGATCCTGTGGATTTCCTTTGCCATATCATGCTTTTTGCTTTGTGTTGTTTTGCTGGGGTTCATCATG
TGGGCCTGCCAGAAAGGCAACATTAGGTGCAACATTTGCATTTGAGTGTATTAGTAATTAAAAACACC
CTTGTTTCTACT

Table S8: Details of Udorn sequences used to assign identity to segments.
Provided in Fasta format.

Udorn genome/mRNA sequences

Segment 1 UTRs from CY003535
>gi|89892490|gb|CY009643.1| Influenza A virus (A/Udorn/72(H3N2))
segment 1, complete sequence
AGCAAAAGCAGGTCAATTATATTCAATATGGAAAGAATAAAAGAACTACGGAATCTGATGTCGCAGTC
TCGCACTCGCGAGATACTAACAAAAACCACAGTGGACCATATGGCCATAATTAAGAAGTACACATCAG
GGAGACAGGAAAAGAACCCGTCACTTAGGATGAAATGGATGATGGCAATGAAATATCCAATTACAGCT
GACAAGAGGATAACAGAAATGGTTCCTGAGAGAAATGAGCAAGGACAAACCCTATGGAGTAAAATGAG
TGATGCCGGGTCAGATCGAGTGATGGTATCACCTTTGGCGGTGACATGGTGGAATAGAAATGGACCAG
TGACAAGTACGGTTCATTATCCAAAAGTCTACAAGACTTATTTTGATAAAGTCGAAAGGTTAAAACAT
GGAACCTTTGGCCCTGTCCATTTTAGAAACCAAGTCAAAATACGCCGAAGAGTTGACATAAACCCTGG
TCATGCAGACCTCAGTGCCAAGGAGGCACAAGATGTAATCATGGAAGTTGTTTTCCCCAATGAAGTGG
GGGCCAGGATACTAACGTCGGAATCACAATTAACAATAACCAAAGAGAAAAAAGAAGAACTCCAAGAT
TGCAAAATTTCTCCTTTGATGGTTGCATACATGTTAGAGAGAGAACTTGTCCGAAAAACGAGATTTCT
CCCAGTTGCTGGTGGAACAAGCAGTGTGTACATTGAAGTGTTACACTTGACTCAAGGAACGTGTTGGG
AACAGATGTACACTCCAGGTGGAGAAGTGAGGAATGACGATGTTGACCAAAGCCTAATTATTGCAGCC
AGGAACATAGTGAGAAGAGCAGCAGTATCAGCAGATCCACTAGCATCTTTATTGGAGATGTGCCACAG
CACACTGATTGGCGGGACAAGGATGGTGGACATTCTTAGGCAGAACCCGACGGAAGAACAAGCTGTGG
ATATATGCAAGGCTGCAATGGGACTGAGGATCAGCTCATCCTTCAGTTTTGGTGGGTTCACATTTAAG
AGAACAAGCGGGTCATCAATCAAAAGAGAGGAAGAAGTGCTTACGGGCAATCTCCAAACATTGAAAAT
AAGGGTGCATGAGGGGTACGAGGAGTTCACAATGGTGGGGAAAAGGGCAACAGCTATACTCAGAAAAG
CAACCAGGAGATTGGTTCAGCTTATAGTGAGTGGAAGGGACGAGCAGTCAATAGCCGAA
GCGATAATTGTAGCCATGGTGTTTTCACAAGAGGATTGCATGATAAAAGCAGTTAGAGGTGACCTGAA
TTTCGTTAACAGGGCAAATCAGCGGTTGAATCCCATGCATCAACTTTTAAGGCATTTTCAGAAAGATG
CGAAAGTGCTTTTTCAGAATTGGGGAATTGAACATATCGACAATGTGATGGGAATGGTTGGAGTATTA
CCAGACATGACTCCAAGCACAGAGATGTCAATGAGAGGAATAAGAGTCAGCAAAATGGGCGTGGATGA
ATACTCCAGCACAGAGAGGGTAGTGGTTAGCATTGATCGGTTTTTGAGAGTTCGAGACCAACGTGGGA
ATGTATTACTATCTCCTGAGGAGGTCAGTGAAACACAGGGGACAGAGAGACTGACAATAACTTACTCA
TCGTCAATGATGTGGGAGATTAATGGCCCTGAGTCAGTGTTGGTCAATACCTATCAATGGATCATCAG
AAACTGGGAAACTGTTAAAATTCAATGGTCTCAGAATCCTACAATGTTGTACAACAAAATGGAATTTG
AGCCATTTCAGTCTTTAGTTCCTAAGGCCATTAGAGGCCAATACAGTGGATTTGTCAGAACTCTATTC
CAACAAATGAGGGATGTACTTGGGACATTTGATACCACCCAGATAATAAAGCTTCTCCCCTTTGCAGC
CGCCCCACCAAAGCAAAGTAGAATGCAGTTCTCTTCATTGACTGTGAATGTGAGGGGATCAGGGATGA
GAATACTTGTAAGGGGCAATTCTCCTGTATTCAACTACAACAAGACCACTAAAAGACTAACAATTCTC
GGAAAAGATGCTGGCACTTTAATTGAAGACCCAGATGAAAGCACATCCGGAGTGGAGTCCGCTGTTTT
GAGAGGATTTCTCATTCTAGGTAAGGAAGATAGAAGATACGGACCAGCATTAAGCATCAATGAACTGA
GTAACCTTGCAAAAGGAGAAAAGGCTAATGTGCTAATTGGGCAAGGAGACGTGGTGTTGGTAATGAAA
CGAAAACGGGACTCTAGCATACTTACTGACAGCCAGACAGCGACCAAAAGAATTCGGATGGCCATCAA
TTAATGTTGAATAGTTTAAAAACGACCTTGTTTCTACT

Segment 2 UTRS from EF467819
>gi|89892487|gb|CY009642.1| Influenza A virus (A/Udorn/72(H3N2))
segment 2, complete sequence
AGCGAAAGCAGGCAAACCATTTGAATGGATGTCAACCCGACTTTACTTTTCTTGAAAGTTCCAGCGCA
AAATGCCATAAGCACCACATTCCCTTATACTGGAGATCCTCCATACAGCCATGGAACAGGAACAGGAT
ACACCATGGACACAGT
CAACAGAACACATCAATATTCAGAAAAAGGGAAGTGGACAACAAACACAGAAACTGGGGCGCCCCAAC
TTAACCCAATTGATGGACCACTACCTGAGGATAATGAGCCAAGTGGATATGCACAAACAGACTGTGTC
CTGGAAGCAATGGCTTTCCTTGAAGAATCCCACCCAGGGATCTTTGAAAACTCGTGCCTTGAAACGAT

GGAAGTCGTTCAACAAACAAGGGTGGACAGACTGACCCAAGGTCGTCAGACCTATGATTGGACATTAA
ACAGAAATCAACCGGCCGCAACTGCATTAGCCAACACTATAGAAGTCTTCAGATCGAATGGTCTAACA
GCTAATGAGTCGGGAAGGCTAATAGATTTCCTCAAGGATGTGATGGAATCAATGGATAAAGAGGAAAT
GGAGATAACAACACACTTCCAAAGAAAAAGAAGAGTAAGAGACAACATGACCAAGAAAATGGTCACAC
AAAGAACAATAGGAAAGAAGAAGCAGAGAGTGAACAAGAGAAGCTATCTAATAAGAGCATTAACATTG
AACACAATGACCAAAGATGCAGAAAGAGGTAAATTAAAGAGAAGAGCTATTGCAACACCCGGGATGCA
AATCAGAGGGTTCGTGTACTTTGTTGAAACTCTAGCTAGGAGCATTTGTGAGAAGCTTGAACAGTCTG
GACTTCCAGTTGGAGGTAATGAAAAGAAGGCCAAACTGGCAAATGTTGTGAGAAAGATGATGACTAAT
TCACAAGACACAGAGCTTTCTTTCACAATTACTGGAGACAATACTAAGTGGAATGAAAATCAAAATCC
TCGAATGTTCCTGGCGATGATTACATATATCACAAAAAATCAACCTGAATGGTTCAGAAACATTCTGA
GCATCGCACCCATAATGTTCTCAAACAAAATGGCGAGACTAGGGAAAGGATACATGTTCGAAAGTAAG
AGAATGAAGCTCCGAACACAAATACCAGCAGAAATGCTAGCAAGCATTGACCTAAAGTATTTCAATGA
ATCAACAAGAAAGAAAATTGAGAAAATAAAGCCTCTTCTAATAGATGGCACAGCGTCATTGAGTCCTG
GAATGATGATGGGCATGTTCAACATGCTAAGTACGGTTTTAGGAGTCTCAATCCTGAATCTTGGGCAA
AAGAAATACACCAAAACAACATACTGGTGGGATGGACTCCAATCCTCTGATGATTTTGCTCTCATAGT
GAATGCACCAAATCATGAGGGAATACAAGCAGGAGTGGATAGATTCTACAGGACCTGCAAGTTAGTCG
GAATCAATATGAGCAAGAAGAAGTCCTATATAAATAGGACAGGAACATTTGAATTCACAAGCTTTTTT
TATCGCTATGGATTTGTAGCCAATTTTAGCATGGAGCTGCCCAGTTTTGGAGTGTCTGGGATTAATGA
GTCAGCTGATATGAGCATTGGAGTAACAGTGATAAAGAACAACATGATAAACAATGACCTTGGACCAG
CAACAGCCCAGATGGCTCTTCAACTGTTCATCAAGGACTACAGATATACATATCGGTGCCACAGAGGA
GACACACAAATTCAGACGAGGAGATCATTCGAGCTAAAGAAGCTGTGGGAGCAAACCCGCTCAAAGGC
AGGACTATTGGTTTCAGATGGAGGACCAAACTTATACAATATCCGGAATCTTCACATCCCTGAAGTCT
GCTTAAAGTGGGAGCTAATGGATGAGGACTATCAGGGAAGACTTTGTAATCCCCTGAATCCATTTGTC
AGCCATAAGGAGATTGAGTCTGTAAACAATGCTGTGGTAATGCCAGCTCATGGTCCAGCCAAGAGCAT
GGAATATGACGCTGTTGCAACTACACACTCCTGGATTCCCAAGAGGAACCGCTCTATTCTCAACACAA
GCCAAAGGGGAATTCTTGAGGATGAACAGATGTATCAGAAGTGCTGCAACCTGTTCGAGAAATTTTTC
CCCAGTAGTTCATACAGGAGACCGGTTGGAATTTCCAGCATGGTGGAGGCCATGGTGTCTAGGGCCCG
GATTGATGCCAGAATTGACTTCGAATCTGGACGGATTAAGAAAGAAGAGTTCGCCGAGATCATGAAGA
TCTGTTCCACCATTGAAGAGCTCAGACGGCAAAAATAATGAATTTAGCTTGTCCTTCATGAAAAAATG
CCTTGTTTCTACT

Segment 3 UTRS FROM CY003533
>gi|89892485|gb|CY009641.1| Influenza A virus (A/Udorn/72(H3N2))
segment 3, complete sequence
AGCAAAAGCAGGTACTGATTCGAGATGGAAGATTTTGTGCGACAATGCTTCAATCCGATGATTGTCGA
GCTTGCAGAAAAGGCAATGAAAGAGTATGGAGAGGATCTGAAAATCGAAACAAACAAATTTGCAGCAA
TATGCACTCACTTGGAGATATGTTTCATGTATTCAGATTTTCATTTCATCAATGAACAAGGCGAGTCA
ATAGTGGTAGAGCTTGATGATCCAAATGCACTGTTAAAGCACAGATTTGAAATAATAGAGGGAAGAGA
CCGCACAATGGCCTGGACAGTAGTAAACAGTATTTGCAACACTACTGGAGCTGAGAAACCGAAGTTTC
TGCCAGATTTGTATGATTACAAGGAGAATAGATTCATCGAGATTGGAGTGACAAGGAGAGAAGTCCAC
ATATACTACCTTGAAAAGGCCAATAAAATTAAATCTGAGAATACACACATCCACATTTTCTCATTCAC
TGGGGAGGAAATGGCCACAAAGGCCGACTACACTCTTGATGATGAAAGCAGGGCTAGGATCAAAACCA
GGCTATTTACCATAAGACAAGAAATGGCCAACAGAGGCCTCTGGGATTCCTTTCGTCAGTCCGAAAGA
GGCGAAGAAACAATTGAAGAAAGATTTGAAATCACAGGAACTATGCGCAGGCTTGCCGACCAAAGTCT
CCCGCCGAACTTCTCCTGCCTTGAGAATTTTAGAGCCTATGTGGATGGATTCGAACCGAACGGCTGCA
TTGAGGGCAAGCTTTCTCAAATGTCCAAAGAAGTGAATGCAAGAATTGAACCTTTTCTGAAGACAACA
CCAAGACCAATCAAACTTCCGGATGGGCCTCCTTGTTTTCAGCGGTCCAAATTCCTTCTGATGGATGC
TTTAAAATTAAGCATTGAAGACCCAAGTCACGAAGGAGAGGGAATACCACTATATGATGCGATCAAGT
GCATGAGAACATTCTTTGGATGGAAAGAACCCTATATCGTCAAACCACACGAAAAGGGAATAAATCCA
AATTATCTGCTGTCATGGAAGCAAGTACTGGCAGAACTACAGGACATTGAAAATGAGGAGAAGATTCC
AAGAACTAAAAACATGAAGAAAACGAGTCAGCTAAAGTGGGCACTTGGTGAGAACATGGCACCAGAGA
AAGTAGACTTTGACAACTGTAGAGACATAAGCGATTTGAAGCAATATGATAGTGACGAACCTGAATTA
